# Predictive value of the serum homocysteine-to-albumin ratio for major adverse cardiovascular events after acute myocardial infarction: a retrospective cohort study

**DOI:** 10.3389/fcvm.2025.1574139

**Published:** 2025-06-20

**Authors:** Jiaping Wang, Jianling Zhou, Na Wang, Meili Fu, Ziwen Song, Ming Hu, Huiyi Wu, Runfeng Sun

**Affiliations:** ^1^ Department of Laboratory Medicine, Donghai People’s Hospital Affiliated to Kangda College of Nanjing Medical University, Lianyungang, China; ^2^ Department of Cardiology, Donghai People’s Hospital Affiliated to Kangda College of Nanjing Medical University, Lianyungang, China

**Keywords:** acute myocardial infarction, homocysteine, albumin, homocysteine-to-albumin ratio, major adverse cardiovascular events, risk stratification, prognostic biomarker

## Abstract

**Background:**

Acute myocardial infarction (AMI) remains a major global health burden, with major adverse cardiovascular events (MACE) significantly affecting long-term prognosis. Although the Global Registry of Acute Coronary Events (GRACE) score is widely used for risk stratification, its complexity limits its clinical utility, especially in resource-limited settings. The serum homocysteine-to-albumin ratio (HAR) is a novel biomarker that combines oxidative stress and inflammatory processes, both of which play key roles in cardiovascular pathophysiology. This study aimed to assess the prognostic significance of HAR for MACE in AMI patients.

**Methods:**

A retrospective cohort study was conducted, including 421 AMI patients admitted to Donghai Hospital from January 2022 to December 2023. Clinical and laboratory data, including homocysteine (Hcy), albumin (Alb), and HAR, were collected. The primary outcome was MACE, defined as cardiovascular mortality, non-fatal myocardial infarction, heart failure, recurrent revascularization, stroke, and severe arrhythmias. Multivariable Cox regression analysis was performed to identify independent predictors of MACE. The predictive performance of HAR was assessed using receiver operating characteristic (ROC) curve analysis and compared with Hcy, Alb, and the GRACE score.

**Results:**

During the 12-month follow-up period, 105 patients (24.9%) experienced MACE. The MACE group had significantly higher Hcy levels (15.7 ± 3.4 vs. 12.6 ± 3.1 μmol/L, *P* < 0.001) and lower Alb levels (34.2 ± 3.9 vs. 38.5 ± 4.2 g/L, *P* < 0.001). HAR was independently associated with MACE (HR = 1.41, 95% CI: 1.20–1.65, *P* < 0.001). ROC analysis demonstrated that HAR (AUC = 0.80) outperformed Hcy (AUC = 0.66) and Alb (AUC = 0.73) in predicting MACE, with predictive accuracy comparable to the GRACE score (AUC = 0.82).

**Conclusions:**

HAR is a strong and independent predictor of MACE in AMI patients. Given its simplicity, cost-effectiveness, and availability, HAR may serve as a valuable biomarker for cardiovascular risk stratification, especially in resource-limited settings. Further prospective studies are warranted to validate these findings and explore potential clinical applications.

## Introduction

Acute myocardial infarction (AMI) is one of the most severe types of coronary heart disease, characterized by a high incidence and high mortality rate. The China Cardiovascular Health and Disease Report 2023 Summary indicates that over the past 20 years, the prevalence and mortality rates of AMI in China have been on the rise (1). Despite advancements in medical therapies that have reduced in-hospital mortality rates for AMI, the incidence of major adverse cardiovascular events (MACE) remains high. MACE includes cardiovascular mortality, nonfatal myocardial infarction, heart failure, recurrent revascularization, and severe arrhythmias, all of which contribute to worsened long-term patient outcomes and impose a substantial socioeconomic burden (2–4). Therefore, effective risk stratification is essential for improving patient prognosis and optimizing healthcare resource allocation.

The Global Registry of Acute Coronary Events (GRACE) score is widely used for AMI prognosis assessment. However, its complexity and dependence on multiple clinical and laboratory parameters limit its applicability, particularly in resource-constrained primary healthcare settings (5, 6). As a result, identifying simpler, cost-effective, and easily accessible biomarkers for predicting MACE is of considerable clinical importance.

Among potential biomarkers, homocysteine (Hcy), a sulfur-containing amino acid, plays a key role in atherosclerosis and thrombosis. Elevated Hcy levels contribute to endothelial dysfunction, inflammation, and platelet aggregation, thereby increasing the risk of cardiovascular complications in AMI patients (7–9). Conversely, albumin (Alb), a critical marker of nutritional status and systemic antioxidative capacity, is essential for maintaining cardiovascular homeostasis. Reduced Alb levels indicate increased systemic inflammation, malnutrition, and impaired endothelial function, all of which are associated with adverse cardiovascular outcomes (10–12). Given their respective roles, integrating these two biomarkers may enhance risk stratification in AMI patients.

The homocysteine-to-albumin ratio (HAR) has recently emerged as a novel biomarker that reflects the combined effects of elevated Hcy and reduced Alb levels. Prior studies have demonstrated that HAR is significantly associated with early neurological deterioration in acute ischemic stroke (13, 14) and serves as a predictor of poor prognosis in patients with chronic heart failure and diabetes mellitus (15, 16). However, its role in AMI and its predictive value for MACE remain insufficiently explored.

This study aimed to evaluate the prognostic significance of HAR in AMI patients and assess its potential utility in predicting MACE. Through a retrospective cohort analysis, this study examined whether HAR could function as a simple yet effective risk stratification tool, thereby facilitating early intervention and improving clinical outcomes in high-risk patients.

## Clinical data and methods

### Study population

This retrospective cohort study was conducted to evaluate the association between the serum homocysteine-to-albumin ratio (HAR) and the risk of major adverse cardiovascular events (MACE) in patients diagnosed with acute myocardial infarction (AMI). The study population comprised patients with AMI who were admitted to the Department of Cardiology at Donghai Hospital, Affiliated Kangda College of Nanjing Medical University, between January 2022 and December 2023.

#### Inclusion and exclusion criteria

Patients were eligible for inclusion if they met the following criteria: diagnosis of acute myocardial infarction (AMI) according to the Fourth Universal Definition of Myocardial Infarction (2018) (17); confirmation of acute coronary artery occlusion via coronary angiography at admission; and age ≥18 years. Although all patients were diagnosed with AMI, coronary artery disease (CAD) was included as a comorbidity to better understand the underlying coronary artery pathology, a key contributor to the development of AMI. The presence of CAD as a comorbidity provides valuable information for assessing cardiovascular risk and the prognosis of AMI patients. Patients were excluded if they met any of the following criteria: (1) severe hepatic dysfunction (e.g., Child-Pugh classification ≥ B) or chronic renal failure (eGFR <30 ml/min/1.73m^2^). (2) Presence of an acute infection or chronic inflammatory disease (e.g., systemic lupus erythematosus, rheumatoid arthritis) within the past 30 days. (3) Diagnosis of malignancy or ongoing antitumor treatment. (4) Pregnancy or lactation. (5) Inability to complete a 12-month follow-up or death from non-cardiovascular causes. (6) Incomplete clinical or laboratory data.

#### Data collection

A total of 421 AMI patients meeting the eligibility criteria were included in the study. Data were retrospectively extracted from hospital medical records and follow-up records. Key data points included: Demographic characteristics: Sex, age, body mass index (BMI), and smoking history. Comorbidities: Presence of hypertension, diabetes, coronary artery disease (CAD), chronic kidney disease (CKD), and history of cerebrovascular disease. Coronary artery lesion characteristics: Findings from coronary angiography, including the number of affected vessels, degree of stenosis, and presence of stent implantation.

#### Baseline treatment

In this study, all patients received standard treatment for acute myocardial infarction (AMI) at the beginning of the study. The treatment included antiplatelet therapy (such as aspirin and clopidogrel), statin medications (such as atorvastatin and simvastatin), and percutaneous coronary intervention (PCI) when necessary. The treatment plan was adjusted based on the clinical condition of the patients. Additionally, the researchers recorded whether patients received folic acid or vitamin B supplementation to lower homocysteine (Hcy) levels. Specifically, 14.3% of patients in the MACE group received folic acid or vitamin B supplementation, while 24.1% of patients in the non-MACE group received the same treatment. All treatment factors were incorporated into the multivariable regression model to control for their potential impact on the occurrence of major adverse cardiovascular events (MACE).

#### Follow-up and study endpoint

Patients were monitored for 12 months post-admission. The primary study endpoint was the occurrence of major adverse cardiovascular events (MACE), defined as cardiovascular death, non-fatal myocardial infarction, rehospitalization for heart failure, repeat revascularization (e.g., percutaneous coronary intervention or coronary artery bypass grafting), stroke, and severe arrhythmias (e.g., ventricular fibrillation or sustained ventricular tachycardia). To ensure data reliability, two cardiologists independently verified and confirmed MACE occurrences. Additionally, the GRACE score was calculated following the 2023 European Society of Cardiology (ESC) Guidelines (2). Ethical approval was obtained from the hospital's ethics committee (Approval No. LJYY2023-02).

### Laboratory tests

Fasting blood samples were collected within 24 h of hospital admission. The following serum biomarkers were analyzed using the AU5800 automated biochemical analyzer (Beckman Coulter, USA): Homocysteine (Hcy), lbumin (Alb). C-reactive protein (CRP). Lipid profile: Low-density lipoprotein cholesterol (LDL-C), high-density lipoprotein cholesterol (HDL-C), triglycerides.Fasting plasma glucose (FPG). Serum creatinine.HAR Calculation: HAR was computed as the ratio of serum Hcy concentration (μmol/L) to serum Alb concentration (g/L).

### Statistical analysis

To ensure comparability between the MACE and non-MACE groups and mitigate potential confounding effects from baseline differences, we applied the following data analysis methods.

### Multivariable Cox regression analysis

Multivariable Cox regression models were applied to evaluate the factors associated with major adverse cardiovascular events (MACE). The models were adjusted for baseline characteristics, including age, sex, diabetes, hypertension, coronary artery disease (CAD), chronic kidney disease (CKD), and other potential confounders. Additionally, since homocysteine (Hcy) levels were higher in the MACE group, folic acid and vitamin B supplementation were incorporated as covariates in the regression model to control for their potential impact on Hcy levels and MACE occurrence. The hazard ratio (HR) for HAR was calculated per 0.1-unit increment, rather than per full unit, to better reflect its clinical relevance within the observed distribution (range: approximately 0.2–0.6; standard deviation: 0.09). This scaling ensures interpretability and avoids overestimation of risk beyond the typical HAR values in our cohort.

The researchers recorded whether patients received folic acid or vitamin B supplementation to lower Hcy and included this factor in the multivariable regression model. The effects of folic acid and vitamin B supplementation on the homocysteine-to-albumin ratio (HAR) and their potential influence on MACE outcomes were considered. Cox regression was used to compare the treatment groups, with the supplementation factor included in the model to control for its potential impact on cardiovascular outcomes. The statistical significance of each covariate was assessed using Wald tests, and a *P*-value of less than 0.05 was considered statistically significant.

#### Stratified analysis

To further explore how different baseline characteristics influence the incidence of MACE, we conducted stratified analyses. Specifically, we stratified the patients by age (<65 years vs. ≥65 years) and the presence or absence of diabetes and hypertension. These stratifications allowed us to evaluate the predictive ability of HAR for MACE within each subgroup.

#### Propensity score matching (PSM)

To further reduce the impact of baseline differences, we employed propensity score matching (PSM). In this process, we performed 1:1 matching based on age and comorbidities (e.g., diabetes and hypertension) with a caliper of 0.1, ensuring that the baseline characteristics of the MACE and non-MACE groups were as comparable as possible.

#### Sensitivity analysis

To assess the robustness of the analysis results, we conducted sensitivity analyses, which included the following: (1) stratification by Age: We divided the patients into two groups (<65 years and ≥65 years) and evaluated the predictive ability of HAR within each age group. (2) Stratification by Comorbidities: We performed subgroup analyses for patients with and without diabetes and hypertension to assess the influence of these conditions on the predictive ability of HAR for MACE. (3) Post-matching Analysis: After applying propensity score matching, we reassessed the predictive value of HAR. We matched 105 pairs of patients and compared the incidence of MACE between the two groups, further confirming the robustness of HAR as a predictive factor. (4) Primary Outcome: The primary outcome of the study was MACE, defined as cardiovascular death, non-fatal myocardial infarction, heart failure, recurrent revascularization, stroke, and severe arrhythmias. All patients were monitored during a 12-month follow-up period, with MACE events independently confirmed by two cardiologists.

#### Statistical methods

All analyses were performed using SPSS software (version 26.0). Data normality was assessed using the Shapiro–Wilk test. For continuous variables, those with a normal distribution were expressed as mean ± standard deviation (SD) and compared using independent *t*-tests. Non-normally distributed variables were expressed as median and interquartile range (IQR) and compared using the Mann–Whitney *U* test. Categorical variables were presented as frequencies and percentages and compared using the chi-square test. The predictive value of HAR was evaluated through Cox regression analysis. We performed ROC curve analysis to evaluate the ability of the GRACE score and the homocysteine-to-albumin ratio (HAR) in predicting major adverse cardiovascular events (MACE). The AUC for each variable was calculated separately to assess its performance as a predictor of MACE. Subsequently, we compared the AUCs of the GRACE score and HAR to further analyze their independent predictive value. The comparison of AUCs was conducted using the DeLong test to determine the statistical significance of the difference between the two models.

## Results

### Baseline clinical characteristics of the study population

A total of 421 patients diagnosed with acute myocardial infarction (AMI) were enrolled in this study and categorized into two groups based on the occurrence of major adverse cardiovascular events (MACE) during the follow-up period. The baseline clinical characteristics of both groups are summarized in Table 1.

**Table 1:** Demographic characteristics, laboratory indicators, comorbidities, and coronary artery lesion grouping results of patients.

Parameter category	Variable	MACE (*n* = 105)	Non-MACE (*n* = 316)	Statistic (*x*^2^*/t*)	*P*-value
Demographic characteristics	Sex (Male/Female)	74 (70.5%)/31 (29.5%)	208 (65.8%)/108 (34.2%)	0.71	0.398
	Age (years)	66.5 ± 9.8	60.8 ± 10.6	2.45	0.015
	Body mass index (kg/m^2^)	27.1 ± 3.6	26.3 ± 3.2	1.43	0.156
	Smoking (Yes/No)	63 (60.0%)/42 (40.0%)	175 (55.4%)/141 (44.6%)	1.02	0.312
Comorbidities	Hypertension	50 (64.1%)	65 (58.6%)	0.51	0.478
	Diabetes mellitus	30 (38.5%)	29 (26.1%)	3.08	0.079
	History of coronary heart disease	46 (59.0%)	38 (34.2%)	8.95	0.003
	Chronic kidney disease	14 (17.9%)	9 (8.1%)	3.95	0.047
	History of cerebrovascular disease	11 (14.1%)	8 (7.2%)	1.93	0.165
Laboratory data	Hcy (μmol/L)	15.7 ± 3.4	12.6 ± 3.1	6.28	<0.001
	Alb (g/L)	34.2 ± 3.9	38.5 ± 4.2	7.07	<0.001
	HAR	0.46 ± 0.11	0.32 ± 0.08	8.19	<0.001
	CRP (mg/L)	13.9 ± 4.8	9.5 ± 3.4	7.34	<0.001
	LDL-C (mmol/L)	3.4 ± 0.8	3.0 ± 0.7	2.07	0.041
	HDL-C (mmol/L)	0.8 ± 0.3	1.1 ± 0.2	7.54	<0.001
	TC (mmol/L)	5.2 ± 1.1	4.9 ± 1.0	1.59	0.114
	TG (mmol/L)	1.8 ± 0.6	1.5 ± 0.4	2.41	0.017
	FBG (mmol/L)	7.8 ± 2.0	6.4 ± 1.9	4.01	<0.001
	Serum creatinine (μmol/L)	94.6 ± 15.1	89.3 ± 14.8	0.0	<0.001
Coronary artery lesions	Number of lesioned vessels	2.8 ± 0.7	2.1 ± 0.6	4.89	<0.001
	Degree of stenosis (Mild/Moderate/Severe)	11/30/64	85/120/111	11.32	0.004
	Stent implantation (Yes/No)	65 (77.4%)/19 (22.6%)	158 (50.0%)/158 (50.0%)	6.52	0.011
Risk score	GRACE score	155.6 ± 25.2	119.5 ± 23.7	7.23	<0.001

No significant differences were observed between the MACE and non-MACE groups in terms of gender distribution (*P* = 0.398), body mass index (BMI) (*P* = 0.156), smoking status (*P* = 0.312), hypertension prevalence (*P* = 0.478), total cholesterol (TC) levels (*P* = 0.114), or history of cerebrovascular disease (*P* = 0.165). Although the prevalence of diabetes mellitus was higher in the MACE group (38.5% vs. 26.1%), the difference did not reach statistical significance (*P* = 0.079).

However, significant differences were observed in age and comorbid conditions. The MACE group exhibited a significantly higher mean age (66.5 ± 9.8 vs. 60.8 ± 10.6 years, *P* = 0.015), along with an increased prevalence of coronary artery disease (59.0% vs. 34.2%, *P* = 0.003) and chronic kidney disease (17.9% vs. 8.1%, *P* = 0.047).

Differences in laboratory parameters were also observed between the groups. The MACE group had significantly elevated levels of homocysteine (Hcy), homocysteine-to-albumin ratio (HAR), C-reactive protein (CRP), fasting plasma glucose (FPG), and serum creatinine (Scr) (all *P* < 0.01). Furthermore, low-density lipoprotein cholesterol (LDL-C) (*P* = 0.041) and triglycerides (TG) (*P* = 0.017) were moderately higher in the MACE group, whereas albumin (Alb) levels were significantly lower (*P* < 0.001).

Regarding coronary artery disease severity, the MACE group exhibited a significantly greater number of diseased vessels (*P* < 0.001) and a higher proportion of severe stenosis cases (*P* = 0.004). In addition, the stent implantation rate was significantly higher in the MACE group (*P* = 0.011). Importantly, The GRACE score, reflecting cardiovascular risk, was significantly higher in the MACE group (*P* < 0.001), indicating an increased likelihood of adverse cardiovascular events.

### Association between HAR and MACE

**Figure 1:**
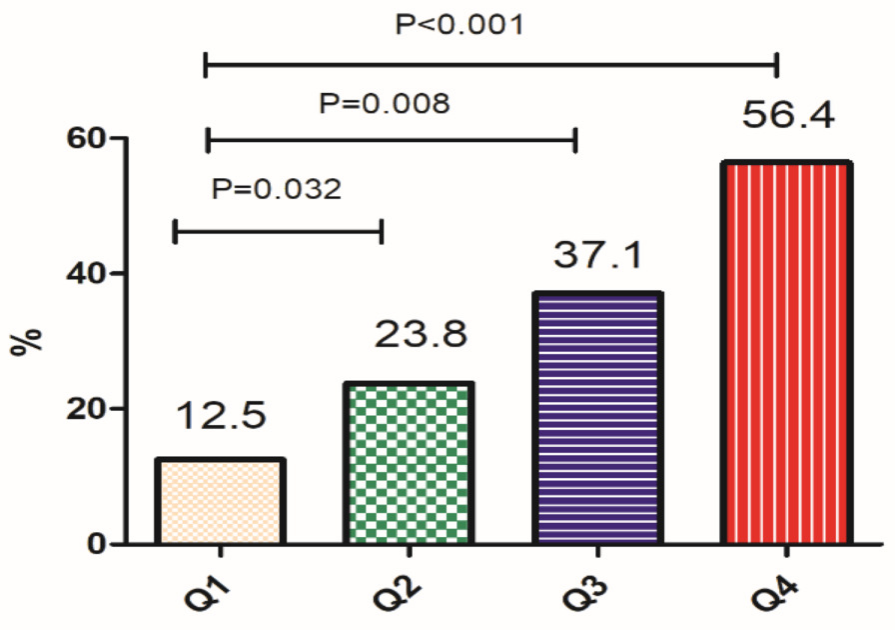
HAR levels and the occurrence of MACE (In the figure: Q1 ≤ 0.28, Q2: 0.29–0.35, Q3: 0.36–0.42, Q4: 0.40–0.43).

To investigate the association between HAR levels and MACE occurrence, the study population was stratified into quartiles (Q1–Q4) based on HAR values. The incidence of MACE was then analyzed across these quartiles Figure 1.

A significant trend was observed, wherein higher HAR levels were associated with an increased risk of MACE. Specifically, patients in the highest HAR quartile (Q4) exhibited a markedly higher incidence of MACE compared to those in the lowest quartile (Q1) (*P* < 0.001). These findings suggest that elevated HAR levels are strongly correlated with adverse cardiovascular outcomes in AMI patients, reinforcing HAR as a potential predictive marker for MACE.

### Correlation between HAR and GRACE score

**Figure 2:**
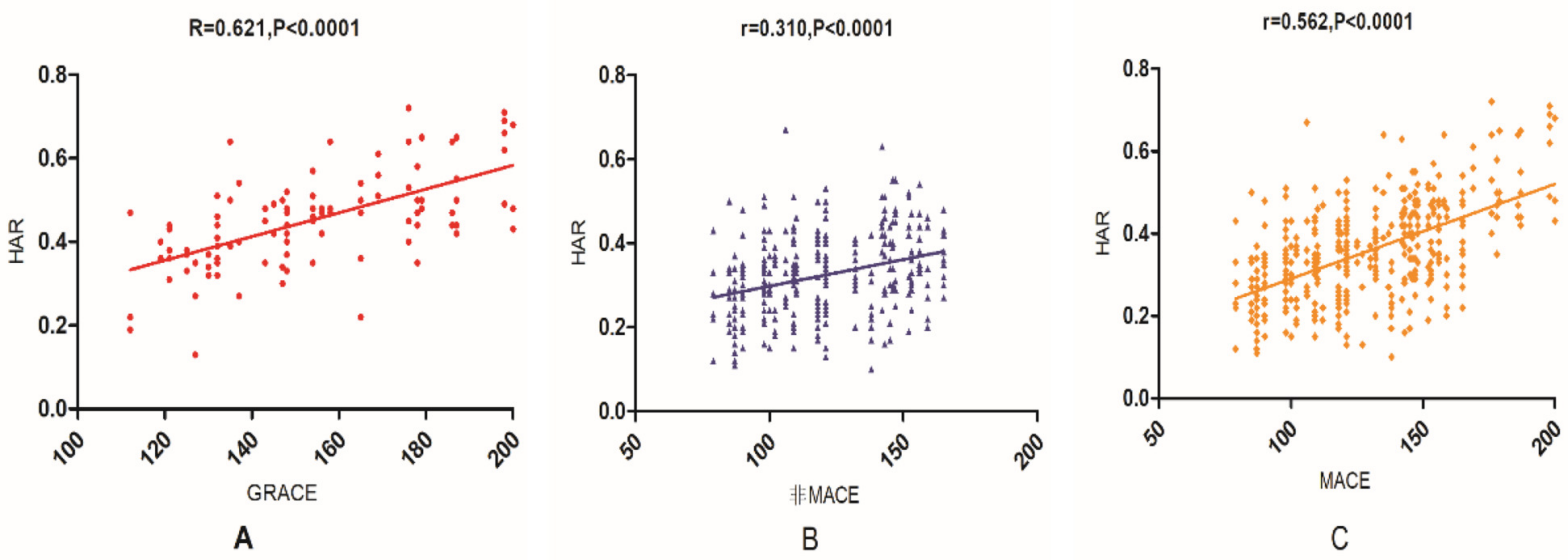
Correlation between HAR and GRACE score in AMI patients (in the figure: **(A)** represents the correlation between HAR and GRACE score in patients with MACE; **(B)** represents the correlation between HAR and GRACE score in patients without MACE; **(C)** represents the correlation between HAR and GRACE score in all AMI patients.).

To further evaluate the prognostic significance of HAR, Spearman correlation analysis was conducted to assess its relationship with GRACE scores in the 421 AMI patients (Figure 2). In the overall AMI cohort, HAR demonstrated a moderate positive correlation with the GRACE score (*γ* = 0.562, *P* < 0.0001).Among patients who experienced MACE, the correlation between HAR and GRACE score was stronger (*γ* = 0.621, *P* < 0.0001), suggesting a closer relationship between these parameters in high-risk individuals.In non-MACE patients, HAR also exhibited a positive correlation with GRACE scores, although the association was weaker (*γ* = 0.310, *P* < 0.0001).

These findings indicate that HAR is closely associated with the GRACE score, particularly in high-risk populations, further supporting its potential utility as a prognostic marker in AMI patients Figure 2.

### Multivariable Cox regression analysis

To determine whether HAR is an independent predictor of MACE, multivariable Cox regression analysis was performed, adjusting for potential confounding variables, including age, sex, CRP levels, diabetes mellitus, hypertension, and lipid profile. The analysis identified elevated Hcy levels, increased HAR, and decreased Alb levels as independent risk factors for MACE (Table 2). Hcy (OR = 1.11, *P* < 0.05): Each 1 μmol/L increase in Hcy was associated with an 11% increased risk of MACE.HAR (*OR* = 1.41, *P* < 0.001): Each 0.1-unit increase in HAR was associated with a 41% increased risk of MACE. In our cohort, HAR values ranged from approximately 0.2–0.6, with a standard deviation of 0.09. Thus, a 0.1-unit increment represents a clinically meaningful change within the observed range. Reporting the hazard ratio per 1.0-unit increase would far exceed the natural distribution of HAR and would substantially overestimate the associated risk. underscoring its strong predictive power. To avoid misinterpretation, it is important to clarify that the reported hazard ratio of 1.41 corresponds to each 0.1-unit increase in HAR. This increment was selected to match the actual variation observed in the study population and to maintain clinical interpretability. We do not suggest a constant risk increase across the entire HAR continuum, but rather highlight that within the observed HAR range, incremental increases are significantly associated with MACE risk. This scaling approach enhances the accuracy and applicability of the risk prediction model.Alb (*OR* = 0.91, *P* < 0.001): Each 1 g/L increase in Alb was associated with a 9% reduction in MACE risk, highlighting its protective role.

**Table 2:** Logistic regression analysis of HCY and HAR for predicting MACE.

Factor	*β* coefficient	SE	Wald	OR	95% CI	*P*-value
Alb	−0.10	0.04	12.50	0.91	0.83–0.97	<0.001
HCY	0.10	0.02	5.76	1.11	1.02–1.20	0.016
HAR	0.34	0.08	18.06	1.41	1.20–1.65	<0.001

These results suggest that HAR serves as a robust independent predictor of MACE, demonstrating a greater predictive effect than Hcy or Alb alone Table 2.

### Stratified analysis

To further investigate the impact of baseline characteristics on the incidence of MACE, stratified analyses based on age and comorbidities were performed. The stratified analysis in Table 2 shows that HAR is a significant predictor of MACE across all subgroups. In younger patients (<65 years), HAR was significantly associated with MACE, and the predictive value was even stronger in patients aged ≥65 years. HAR also demonstrated stronger predictive power in diabetic patients (HR = 1.32, *P* = 0.008) compared to non-diabetic patients, where the association was weaker and not statistically significant. Similarly, HAR was a strong predictor in hypertensive patients (HR = 1.35, *P* = 0.004), but its predictive value was weaker in those without hypertension. Overall, these findings support the use of HAR as a reliable cardiovascular risk marker, particularly in higher-risk groups such as the elderly, diabetic, and hypertensive individuals Table 3.

**Table 3:** Results of stratified analysis by age and comorbidity.

Subgroup	Hazard ratio (HR)	95% CI	*P*-value
Age (<65 years)	1.25	1.05–1.47	0.015
Age (≥65 years)	1.40	1.20–1.64	0.002
Diabetes (Yes)	1.32	1.08–1.61	0.008
Diabetes (No)	1.20	0.99–1.45	0.073
Hypertension (Yes)	1.35	1.12–1.63	0.004
Hypertension (No)	1.18	0.97–1.43	0.095

### Propensity score matching (PSM)

To further reduce the impact of baseline differences, we performed propensity score matching (PSM), matching 105 pairs of patients based on age and comorbidities (diabetes and hypertension). After matching, HAR remained a strong predictor of MACE (HR = 1.37, 95% CI: 1.15–1.63, *P* = 0.001). The incidence of MACE was higher in the group with higher HAR levels compared to the lower HAR group, further confirming HAR's predictive value.

### Predictive performance of HAR, and GRACE scores for MACE

To further assess the predictive utility of HAR, a receiver operating characteristic (ROC) curve analysis was conducted to evaluate the performance of HAR, and GRACE scores in predicting MACE.

The AUC values for the GRACE score and the homocysteine-to-albumin ratio (HAR) in predicting major adverse cardiovascular events (MACE) were calculated separately. The AUC for the GRACE score was 0.82 (95% CI: 0.7612–0.8788), while the AUC for HAR was 0.80 (95% CI: 0.7412–0.8588). Although the AUC of the GRACE score was slightly higher than that of HAR, there was no significant difference between the two (*P* = 0.12).

**Figure 3:**
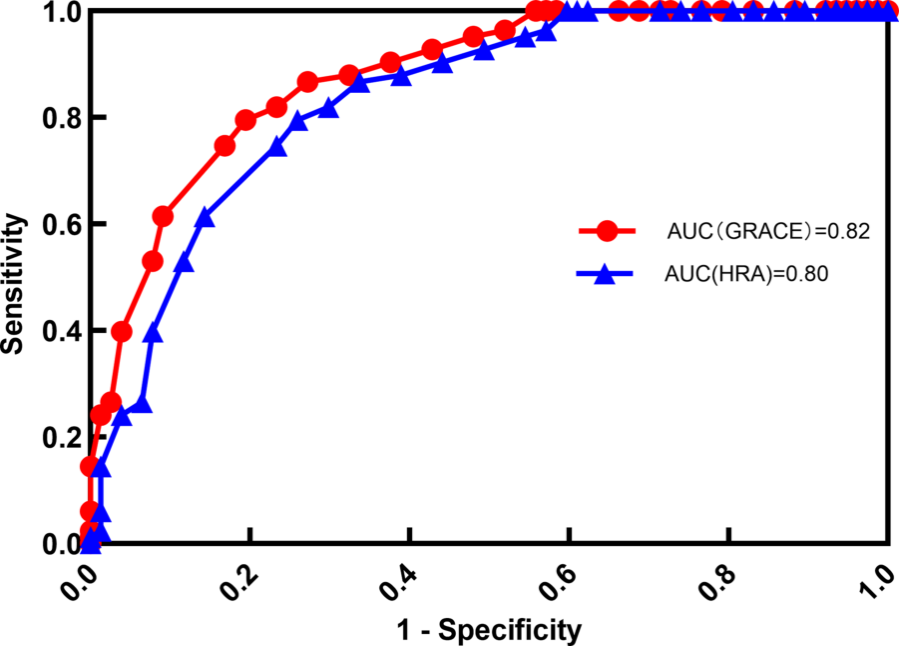
ROC curves for predictive performance of HCY and HAR on MACE.

The sensitivity of the GRACE score and HAR was 75.8% and 72.9%, respectively, while the specificity was 77.2% and 78.6%, respectively. As illustrated in Figure 3, Table 4.

**Table 4:** Predictive performance analysis of HCY and HAR for MACE.

Item	AUC	Optimal cutoff	SE	Sensitivity (%)	Specificity (%)	Likelihood ratio (LR+)	95% CI	*P*-value
GRACE	0.82	139.8	0.02	75.8	77.2	3.33	0.78–0.86	<0.001
HAR	0.80	0.36	0.03	72.9	78.6	3.41	0.76–0.84	<0.001

### Primary outcome

The primary outcome, MACE, occurred in 105 patients (24.9%) during the 12-month follow-up period. The incidence of MACE was significantly higher in patients with elevated HAR levels compared to those with lower HAR levels. The association between HAR and MACE was consistent across all stratified and sensitivity analyses.

## Discussion

Homocysteine (Hcy) is an independent risk factor for cardiovascular diseases (CVDs), contributing to disease progression through multiple pathological mechanisms. It promotes atherosclerosis via oxidative stress, endothelial dysfunction, inflammation, and thrombosis (18, 19). Elevated Hcy induces reactive oxygen species (ROS) production, leading to vascular endothelial damage and low-density lipoprotein (LDL) oxidation, thereby accelerating atherosclerotic progression (20). Additionally, Hcy interacts with endothelial cell thiol groups, reducing nitric oxide (NO) synthesis and impairing vascular relaxation, further exacerbating endothelial dysfunction (21). In the present study, patients who experienced major adverse cardiovascular events (MACE) had significantly higher Hcy levels than those who did not, suggesting that Hcy elevation plays a pivotal role in adverse cardiovascular outcomes following acute myocardial infarction (AMI).

Albumin (Alb), a key indicator of systemic inflammation and nutritional status, has also been associated with CVD prognosis. Low Alb levels are linked to poor cardiovascular outcomes, likely due to increased inflammation, diminished antioxidant capacity, and a hypercoagulable state (22, 23). As an antioxidant, Alb neutralizes ROS, reducing oxidative stress-induced endothelial dysfunction. Furthermore, hypoalbuminemia reflects nutritional deficits, compromising the body's resilience to acute physiological stress (24). The findings of this study corroborate these associations, as patients in the MACE group exhibited significantly lower Alb levels than those in the non-MACE group. These results reinforce the hypothesis that hypoalbuminemia contributes to adverse cardiovascular outcomes after AMI, likely by intensifying inflammation and oxidative stress (25).

Given the distinct but interrelated roles of Hcy and Alb in CVD pathogenesis, their combined measure—the homocysteine-to-albumin ratio (HAR)—has been proposed as a more comprehensive risk indicator. The present study is the first to systematically evaluate HAR in AMI patients. The results demonstrated that HAR exhibited greater predictive value than Hcy or Alb alone, highlighting its potential as a clinically relevant biomarker.

This finding suggests that HAR integrates multidimensional pathophysiological changes, making it a robust tool for risk stratification.

This study demonstrated that HAR is a significant predictor of MACE in a cohort of AMI patients, with stronger associations observed in specific subgroups, such as older patients and those with comorbid conditions like diabetes and hypertension. Despite significant baseline differences, particularly in age and comorbidities, our results remained consistent across all stratified and sensitivity analyses. These differences were addressed using statistical methods, including multivariable Cox regression, propensity score matching (PSM), and stratified analysis by age and comorbidities, ensuring the validity of our findings. Higher HAR values correlated with a greater incidence of MACE, as confirmed by multivariable Cox regression analysis. Moreover, receiver operating characteristic (ROC) curve analysis demonstrated that HAR provided superior predictive accuracy compared to Hcy or Alb alone, reinforcing its clinical applicability as a risk stratification biomarker. Although folic acid and vitamin B supplementation may affect homocysteine (Hcy) levels, only a small proportion of patients in our study received such treatment. To mitigate potential confounding, we included supplementation status as a binary covariate in the multivariable regression model. Importantly, HAR consistently remained a strong and independent predictor of MACE across all stratified and sensitivity analyses, indicating that any potential multicollinearity had a negligible impact on the overall robustness of the results.

To further validate HAR, its relationship with the Global Registry of Acute Coronary Events (GRACE) score was assessed. A strong positive correlation was observed, with HAR achieving an area under the curve (AUC) of 0.80, comparable to the GRACE score (AUC = 0.82) and within the established AUC range for GRACE in predicting MACE (0.73–0.82) (6, 7). It should be noted that HAR and the GRACE score are not directly comparable. The GRACE score is a risk assessment tool based on multiple clinical parameters (such as age, heart rate, blood pressure, etc.) and is widely used for short-term risk assessment in patients with acute coronary syndrome. In contrast, HAR is a predictive factor based on biomarkers, reflecting the ratio of homocysteine to albumin, offering a different biological perspective.

Although the risk factors assessed by the two tools differ, HAR and the GRACE score can complement each other. By reflecting biological pathways such as inflammation and oxidative stress, HAR may provide additional information for risk assessment, particularly in specific subgroups (e.g., patients with diabetes or hypertension). Future studies could explore how to combine HAR with traditional clinical scores (such as the GRACE score) to further improve the accuracy of cardiovascular risk assessment. Although all patients in this study were diagnosed with acute myocardial infarction (AMI), coronary artery disease (CAD) was included as a comorbidity to provide a more comprehensive understanding of the baseline coronary artery pathology. CAD is the primary underlying cause of AMI, and its inclusion as a comorbidity enables more accurate risk stratification while helping to contextualize the role of the homocysteine-to-albumin ratio (HAR) as a predictor of major adverse cardiovascular events (MACE). By assessing both CAD and HAR, we gain insights into both the structural pathology and the biological processes contributing to adverse cardiovascular outcomes.

Given the association between high HAR and MACE, early therapeutic interventions aimed at reducing oxidative stress and inflammation may mitigate cardiovascular risk. Strategies such as folic acid or vitamin B supplementation to lower Hcy levels and nutritional interventions to improve Alb levels could potentially enhance long-term cardiovascular outcomes (26, 27). Given its strong predictive capacity, HAR may be a valuable tool in primary care settings for identifying high-risk individuals and facilitating timely medical intervention.

The homocysteine-to-albumin ratio (HAR) emerges as a promising biomarker for predicting cardiovascular events in clinical practice, particularly in high-risk populations. Despite significant baseline disparities between the MACE and non-MACE groups (e.g., age and comorbidities), our analytical approach—incorporating multivariable Cox regression, propensity score matching (PSM), and stratified analyses—robustly demonstrated that HAR retained its independent predictive value for MACE after rigorous adjustment for these confounders. This finding holds critical clinical relevance for high-risk subgroups such as elderly patients and individuals with diabetes or hypertension, where early risk stratification using HAR may facilitate timely therapeutic interventions and personalized management strategies.

While the findings indicate that HAR performs comparably to the GRACE score, these two risk stratification tools may serve complementary roles. The GRACE score is primarily based on clinical and physiological parameters, whereas HAR reflects inflammation and oxidative stress, capturing additional aspects of cardiovascular risk.

### Study limitations

Despite these promising results, The study's limitations include its retrospective design and single-center nature, which may limit the generalizability of the findings. Although we adjusted for significant baseline differences in age and comorbidities using multivariable Cox regression and propensity score matching (PSM), potential residual confounding factors cannot be fully excluded. Furthermore, the relatively short follow-up period of 12 months may not be sufficient to capture long-term cardiovascular outcomes. Future studies should focus on validating these findings in larger, more diverse populations across multiple centers to assess the generalizability of HAR as a cardiovascular risk marker. Additionally, prospective studies with longer follow-up periods are needed to evaluate the long-term prognostic value of HAR in predicting cardiovascular events. Mechanistic studies exploring the biological pathways linking HAR to cardiovascular outcomes, particularly its role in inflammation, oxidative stress, and endothelial dysfunction, would provide valuable insights into its clinical utility. Clinical trials examining interventions aimed at modulating HAR levels could also provide important evidence of its therapeutic potential.

This study found that, although homocysteine (Hcy) levels were elevated in the MACE group, folic acid and vitamin B supplementation may help reduce Hcy levels. While existing literature has reported the potential cardiovascular benefits of such supplementation, its specific impact on the occurrence of major adverse cardiovascular events (MACE) remains unclear and warrants further investigation.

We included folic acid/vitamin B supplementation in the multivariable regression model to control for its potential effect on Hcy levels and cardiovascular events. However, due to limitations in sample size and treatment biases, this study was unable to fully eliminate all confounding factors. Therefore, further research is needed to explore this issue more comprehensively.

Although this study suggests that folic acid/vitamin B supplementation may provide benefits for AMI patients, the underlying mechanisms remain unclear. Future prospective studies with larger sample sizes are necessary to further validate its preventive effects on cardiovascular adverse events.

## Conclusion

This study is the first to establish HAR as an independent predictor of MACE following AMI and to validate its prognostic utility. By capturing systemic inflammation and oxidative stress, HAR provides a novel and practical approach to risk stratification in AMI patients. Given its ease of measurement and broad applicability, HAR represents a valuable tool for early risk assessment and precision medicine strategies, ultimately contributing to improved long-term cardiovascular outcomes.

## Data Availability

The raw data supporting the conclusions of this article will be made available by the authors, without undue reservation.
